# Fabrication and Characterization of Low Methoxyl Pectin/Gelatin/Carboxymethyl Cellulose Absorbent Hydrogel Film for Wound Dressing Applications

**DOI:** 10.3390/ma12101628

**Published:** 2019-05-17

**Authors:** Pensak Jantrawut, Juthamart Bunrueangtha, Juthamart Suerthong, Nutthapong Kantrong

**Affiliations:** 1 Department of Pharmaceutical Sciences, Faculty of Pharmacy, Chiang Mai University, Chiang Mai 50200, Thailand; Juthamart.bun@gmail.com (J.B.); st.juthamas@gmail.com (J.S.); 2 Cluster of Research and Development of Pharmaceutical and Natural Products Innovation for Human or Animal, Chiang Mai University, Chiang Mai 50200, Thailand; 3 Department of Restorative Dentistry, Faculty of Dentistry, Khon Kaen University, Khon Kaen 40002, Thailand; 4 Oral Biology Research Unit, Faculty of Dentistry, Khon Kaen University, Khon Kaen 40002, Thailand

**Keywords:** hydrogel, film, povidone iodine, low methoxyl pectin, gelatin, carboxymethyl cellulose

## Abstract

In this study, hydrogel films composed of low methoxyl pectin (LMP), gelatin, and carboxymethyl cellulose (CMC) were fabricated. Glycerin was used as a plasticizer while glutaraldehyde (Glu) and calcium chloride (CaCl_2_) were used as crosslinking agents in film preparation. Hydrogel films were morphologically characterized and evaluated for mechanical properties. In addition, the investigations for fluid uptake ability, water retention capacity, water vapor transmission rate, and integrity value of the invented films were performed. The results showed that F-Glu-Ca-G30 film demonstrated superior properties when compared to other prepared films. It demonstrated a high percentage of elongation at break (32.80%), fluid uptake ability (88.45% at 2 h), water retention capacity (81.70% at 2 h), water vapor transmission rate (1889 g/m^2^/day), and integrity value (86.42%). F-Glu-Ca-G30 film was subsequently selected for 10% w/w povidone iodine (PI) loading and tested for anti-*Staphylococcus aureus* activity using an agar diffusion assay. Notably, F-Glu-Ca-G30-PI film demonstrated a dramatic ability to inhibit microbial growth, when compared to both a blank film and iodine solution control. Our LMP/gelatin/CMC hydrogel film promises to be an effective dressing material with high fluid absorption capacity, fluid holding ability, and water vapor transmission rate. Incorporation of antibiotics such as povidone iodine into the films conferred its antimicrobial property thereby highlighting its potential dermatological use. However, further clinical studies of the application of this hydrogel film as wound dressing material is recommended.

## 1. Introduction

Currently, numerous wound dressing materials are available and are also being investigated [1,2,3]. The desirable wound dressing may therefore serve among various purposes, such as providing moisture and occlusion, and protect from infections and contamination [4,5,6]. Among wound dressings, dressings casted from hydrogels, sometimes known as hydrocolloid dressings, have become the first major advancement and are used in the management of moist wounds [7], a clinical situation during which proper wound healing is promoted by dressing materials capable of maintaining a moist environment. When in contact with wound exudates, hydrogel has an immediate reaction to form gels and high fluid absorption occurs via strong hydrophilic gel formation [8]. In order for a hydrogel to maintain its three-dimensional structure, the hydrophilic polymer chains are cross-linked either by covalent or non-covalent bonds, i.e., electrostatic, hydrophobic, or van der Waals interactions [9]. In fact, the fluid handling capacity of hydrogel dressings depends on several factors, such as type of dressing materials, its inherent physicochemical properties, as well as the design of the dressing [10].

Hydrogels can be formed by dissolving natural polymers, such as chitosan, gelatin, collagen, pectin, and carboxymethyl cellulose (CMC) with water. Interestingly, hydrophilic synthetic polymers can also be dissolved in water to form hydrogels [11,12]. Gelatin, CMC, as well as pectin, have been used in a number of hydrogel formulations as wound dressings [13,14]. There are reports suggesting that gelatin is used for hemostasis in bleeding wounds [15]. CMC exhibits a high water bonding affinity with excellent skin compatibility and is able to maintain an optimal moist environment in the wound region [16,17]. Low methoxyl pectin (LMP) also has several unique properties that have enabled it to be used as a matrix with its distinction in water absorption and retention properties, hence known as superabsorbents [18,19,20]. Furthermore, crosslinked polymers derived from LMP can be used as a matrix for entrapment and/or delivery of a variety of drugs [21], including metronidazole [22]. Thus, our main goal of this study was to seek an optimal formulation of hydrogel film used to deliver povidone iodine. In this report, we have focused on the development of an in-house film by using three hydrophilic polymers including LMP, gelatin, and CMC for fabricating the hydrogel film. We hope that the combination of these hydrophilic polymers’ properties might lead to the invention of hydrogels with a potential use as biomaterials. After preparation, hydrogel films were characterized, investigated for their fluid absorption properties, and further loaded with povidone iodine to demonstrate their potential application in drug delivery. An antibacterial activity against *Staphylococcus aureus* (*S. aureus*), a common pathogen capable of causing clinically significant infection in contaminated wounds, was tested in vitro.

## 2. Materials and Methods

### 2.1. Materials and Reagents Used 

Low methoxyl pectin (LMP; Degree of esterification = 29%) was purchased from Cargill, Saint Germain, France. Carboxymethyl cellulose (CMC) was purchased from CP Kelco Oy, Aanekoski, Finland. Gelatin was purchased from McGarrett, Bangkok, Thailand. Calcium chloride (CaCl_2_) was purchased from RCI Labscan Ltd., Bangkok, Thailand. Glutaraldehyde (Glu) 25% was purchased from AppliChem GmbH, Darmstadt, Germany. Povidone iodine (PI) was purchased from Qchemical Co. Ltd., Bangkok, Thailand. Deionized water served as the solvent for preparing film solution. 

### 2.2. Preparation of Hydrogel Films

Hydrogel films were produced by separately preparing 1% w/w each of LMP, gelatin, and CMC in deionized water after which they were mixed, heated to 60 ± 0.5 °C and held for 1 h, then cooled to 35 ± 0.5 °C. At this temperature, 0–40% w/w glycerin and 0.3% w/w glutaraldehyde (Glu) were added to the solution under thorough and continuous mixing (500 rpm) to crosslink the gelatin and form hydrogel. All hydrogels were degassed and subsequently casted by pouring approximately 300 g onto the polycarbonate rectangular templates (17 cm length × 8.5 cm width). The hydrogel film was dried in an oven at 40 ± 2 °C for 48 h. Since calcium ions have been reported as crosslinkers in earlier studies for the preparation of LMP films [22,23], an optimized volume of 3% w/w CaCl_2_ was poured on LMP/gelatin/CMC films in the templates. After 24 h, crosslinked films were taken out, and additionally washed with deionized water and then air-dried at room temperature for 24 h. After the drying step, translucent LMP/gelatin/CMC films were obtained. Films with the presence of any defective surface or trapped air bubbles were not used for the study. LMP/gelatin/CMC films were originally prepared with different film formulations with varying glycerin content as shown in Table 1. The hydrogel film that demonstrated better tensile and absorption properties was selected to load povidone iodine (PI) as a model drug and investigated for in vitro antibacterial activity. We chose PI as our model drug since it is one of the most common aseptics used and can be easily incorporated into hydrogel films. Briefly, full-strength PI solution was simultaneously incorporated into the LMP/gelatin/CMC solution to achieve a concentration of 10% w/w and then the steps described above were followed.

### 2.3. Hydrogel Film Characterizations

#### 2.3.1. Morphological Analysis Using Scanning Electron Microscopy

Morphological study of hydrogel films was performed on a Scanning Electron Microscopy (SEM) (JSM-5410LV, JEOL Ltd., Peabody, MA, USA) at 10 kV. Film samples were examined for cross section and surface characteristics by affixing to aluminum stubs with double-sided cellophane adhesive tape and sputter-coated with a layer of gold prior to imaging at 100× and 200× magnification levels. 

#### 2.3.2. Film Thickness 

The average thickness of hydrogel films was determined using a thickness gauge (GT-313-A, Gotech Testing Machines Inc., Taichung, Taiwan). Five random measurements were taken on each film. The average of the five values and their standard deviation (SD) of individual films were calculated. Thickness measurements were performed in triplicate. 

#### 2.3.3. Tensile Strength

Tensile strength of hydrogel film was evaluated using a texture analyzer TX.TA plus (Stable Micro Systems, Surrey, UK), similar to a previous study [23]. Under laboratory investigation, the hydrogel films were cut into a rectangle shape of 2 × 7 cm^2^. The film sample was clamped between two tensile grips for film testing and the initial gauge length was set at 5 cm. The film was pulled using a crosshead speed of 2 mm/min. During the stretching, force (N) and elongation at break (mm) were recorded. At least five repeats were carried out for each hydrogel film formulation and average tensile strength, percentage of elongation at break, and Young’s modulus values were calculated. 

#### 2.3.4. Fluid Uptake Ability

The fluid uptake ability of hydrogel films was determined using a gravimetric method [24]. Initially, hydrogel films were cut into 3 × 3 cm^2^ pieces and their dry weights (Wd) were measured. Each sample was immersed in a vial containing 15 mL phosphate buffer saline (PBS) pH 7.4 and incubated at 37 °C. At certain intervals, the swollen films were withdrawn from PBS. The wet weight of the swollen films (Ws) was measured after the removal of excess surface PBS by gently blotting with a filter paper. Weights of hydrogel films were recorded until the swelling equilibrium was reached. The equilibrium of fluid content was defined by the following equation: equilibrium of fluid content (%) = (Ws−Wd)/Ws × 100. These tests were carried out in triplicate.

#### 2.3.5. Water Retention Capacity

The water-conserved capacity of hydrogel film was evaluated by a water retention test. Films were immersed in deionized water for 24 h. The swollen films were then wiped with a filter paper to remove surface water, then the initial wet weight (W0) was measured and they were placed in open mouth dishes at room temperature in 60% relative humidity. After a period of 24 h, specimens were taken out and weighed (Wt). Water retention capacity (%) was defined by the following equation: water retention capacity (%) = Wt/W0 × 100. These tests were carried out in triplicate.

#### 2.3.6. Water Vapor Transmission Rate

Water vapor transmission rate (WVTR) of hydrogel film was determined by adopting a standard method [25]. Briefly, each film was mounted on the mouth of a cylindrical glass vial (34 mm diameter) containing 10 mL water. The material was fastened using Teflon tape across the edges to prevent any water vapor loss through the boundary and kept in a 37 °C incubator in with 35% relative humidity. Evaporation of water through the film was determined by periodic weighing. Weight changes indicated the loss of water. The assembly was weighed at regular intervals of time for 48 h and a weight loss versus time plot was constructed. From the slope of the plot, WVTR was calculated by the following equation: WVTR (g/m^2^/day) = (slope × 24)/A, where A is the test area of the sample in m^2^. Experiments were performed in triplicate and the average values were calculated.

#### 2.3.7. Integrity Value

Samples were cut into a 3 × 3 cm^2^ dimension and the initial weight (Wi) was measured. Each sample was placed in a glass bottle containing 30 mL PBS and agitated on a shaker (Eberbach Co., Ann Arbor, MI, USA) at low speed for 24 h. Then, samples were removed, transferred to a metal pan, and dried for 24 h in a 65 °C oven (Memmert GmbH Co., KG, Schwabach, Germany). Dried weight (Wd) was then utilized for calculating the integrity value using the following equation: integrity value (%) = Wd/Wi × 100 [26]. Experiments were performed in triplicate and the average values were calculated.

### 2.4. Test of Antibiotic-Containing Hydrogel Films’ Ability to Confer an Antimicrobial Property

*S*. *aureus* ATCC 25923 DMST 8840 strain was obtained from our bacterial culture collections and grown in tryptic soy agar (HiMedia, Mumbai, India). Isolated bacterial colonies were subsequently inoculated into tryptic soy broth (Sigma-Aldrich, St. Louis, MO, USA) and cultured at 37 °C under an aerobic atmosphere. An overnight culture of *S*. *aureus* was determined for the optical density at 600 nm wavelength (OD600) using a spectrophotometer (Beckman Coulter, Fullerton, CA, USA) prior to the preparation of bacterial stock for spreading on a tryptic soy agar plate.

Povidone iodine-containing hydrogel films were tested for their antibacterial activity using an agar diffusion method. Briefly, F-Glu-Ca-G30 hydrogel films containing 10% w/w povidone iodine were placed on prepared bacterial agar plates. Blank F-Glu-Ca-G30 films were used as a negative control. In addition, 10 μL iodine solution and DMSO (RCI Labscan Ltd., Bangkok, Thailand) were loaded separately on Whatman® antibiotic assay discs (GE Healthcare, Pittsburgh, PA, USA) and served as a positive control and a vehicle control, respectively. Our test samples were kept in a 37 °C incubator under aerobic cultivation overnight until bacterial lawns were clearly visible. After a 12 h incubation period, the diameter of an inhibition zone was determined using a Mitutoyo® Digimatic caliper (Mitutoyo Corporation, Kanagawa, Japan). Three independent experiments were performed in triplicate. 

### 2.5. Statistical Analysis 

All data were presented as mean ± SD. One-way ANOVA was used to determine the significant difference at which statistical significance was reported when the *p*-value was less than 0.05. Statistical analysis was performed using SPSS software version 16.0 (SPSS Inc., Chicago, IL, USA).

## 3. Results and Discussion

### 3.1. Preparation and Morphology of Hydrogel Films

LMP/gelatin/CMC hydrogel films were prepared by a solvent casting method using Glu and CaCl_2_ as crosslinkers. Generally, gelatin as well as CMC alone can physically form hydrogels by physical crosslinking in water; however, they are easily broken resulting in a significant limitation of their biomedical applications [27,28]. To increase their mechanical properties, gelatin and CMC gels can be covalently crosslinked by adding a small chemical such as Glu [29,30]. When Glu is added, there are numerous hydroxyl and amino groups generated on the macromolecular chains of gelatin and CMC that may react with Glu. As a consequence, the structure of crosslinked materials becomes more complex. They are considered to be interpenetrated-interconnected networks [30]. Remarkably, LMP can be crosslinked by CaCl_2_ in this study. It is well known that divalent cations such as Ca^2+^ can induce gelation of LMP [31]. When CaCl_2_ is introduced into a solution of pectin, gel is formed immediately. The structure of crosslinking is explained by the “egg-box” model, based upon the linkage conformations of the galacturonic residues [20,31].

**Figure 1:**
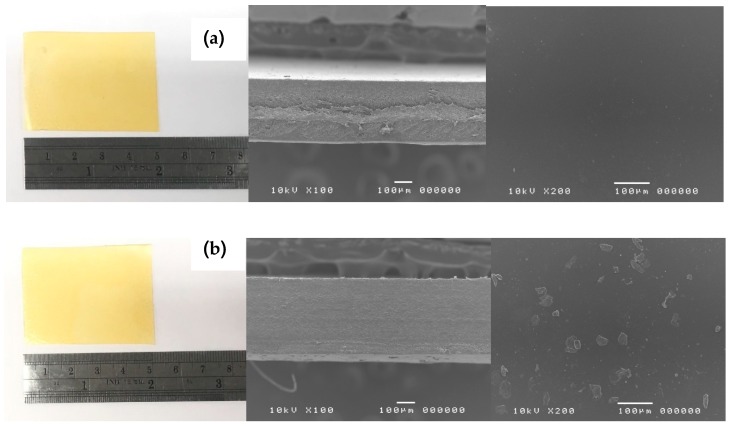
The gross appearance (left) and scanning electron images of cross-section at 100× (middle) and surface at 200× (right) of F-Glu (**a**) and F-Glu-Ca-G30 (**b**) hydrogel films.

From Figure 1, visual examination of all LMP/gelatin/CMC hydrogel films did not show any significant difference. All hydrogel films were translucent, smooth, with an average thickness of 300 to 400 µm without pores and cracks on the surface. However, a SEM image of F-Glu-Ca-G30 hydrogel film showed a heterogeneous structure with the presence of CaCl_2_ crystals on the surface of the film, while a SEM image of F-Glu hydrogel film which had only Glu as a crosslinker showed a homogenous surface, as shown in Figure 1. These CaCl_2_ crystals were also found in all hydrogel films using CaCl_2_ as a secondary crosslinking agent due to CaCl_2_ excess which led to recrystallization of CaCl_2_ during solvent evaporation. Thickness of hydrogel films was also measured using a thickness gauge and we found a consistency of thickness between different groups of prepared hydrogel films, ranging between 0.30 and 0.37 mm thickness. Notably, the thickness of hydrogel film was slightly increased when glycerin was added but there was no significant difference among the thickness of all hydrogel films, as shown in Table 2. 

### 3.2. Mechanical Properties of Hydrogel Films

Tensile strength, elongation at break, and Young’s modulus of hydrogel films consisting of different compositions are shown in Table 2. Between the hydrogel films using Glu (F-Glu) and Glu with CaCl_2_ (F-Glu-Ca) as crosslinkers, F-Glu-Ca film was mechanically stronger as indicated by significantly high values of tensile strength (853.06 MPa) and Young’s modulus (56,781.02 N/cm^2^) with low value of elongation (3.22%). The tougher and rigid texture of F-Glu-Ca film was thought to be due to the presence of CaCl_2_ which was the second crosslinker in the film formulation. For an ideal wound dressing material, wound dressing films must have a high elongation at break, high tensile strength, and low Young’s modulus providing that wound dressings are required to be durable and stress resistant for their application and handling purposes [32,33,34]. Hence, glycerin, a commonly employed chemical in hydrogel preparation, was used in this study as a plasticizer to increase the film flexibility. Results showed that F-Glu-Ca-G40 film exhibited significantly higher percentage of elongation (36.25%) and lower Young’s modulus (2835.37 N/cm^2^) than those of F-Glu-Ca-G10 and F-Glu-Ca-G20 (p < 0.05). Generally, it seems that the increase of glycerin concentration tends to reduce tensile strength and Young’s modulus, while elevating the percentage of film elongation. This is mainly caused by the ability of glycerin to enhance flexibility and lessen intermolecular forces along the polymer chains [35]. In our study, F-Glu-Ca-G40 exhibited slightly lower on tensile strength and Young’s modulus with higher percentage of elongation when compared to F-Glu-Ca-G30, and no significant difference was found between them. This result suggested that addition of 30% w/w glycerin was enough to generate a flexible LMP/gelatin/CMC hydrogel film, by improving its mechanical properties. Another study showed that film dressings composed of lactic acid and caproic acid copolymer exhibited 50% elongation, rendering them being ideal for applying on wounds [36]. Thus, further improvement on the percentage of film elongation in this study could attribute to an achievement of the maximal physical property.

### 3.3. Fluid Uptake Ability, Water Retention Capacity, Water Vapor Transmission Rate, and Integrity Value of Hydrogel Films

**Figure 2:**
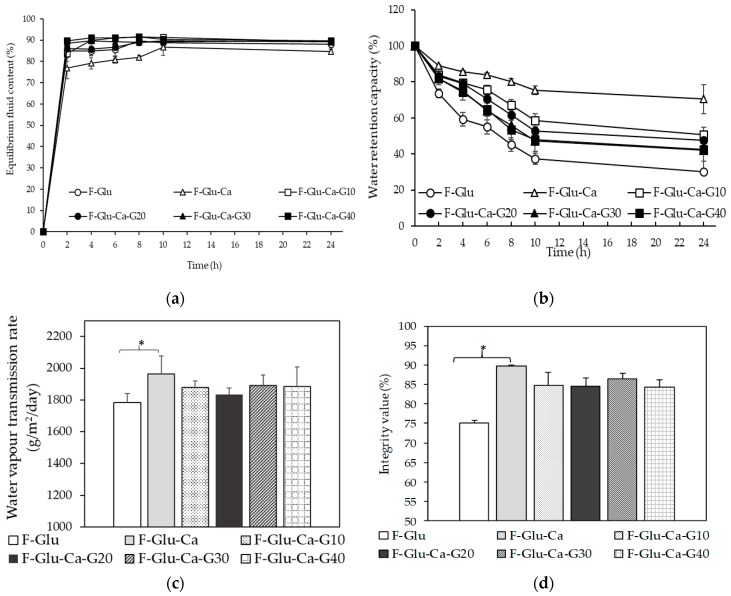
Fluid uptake ability (**a**), water retention capacity (**b**), water vapor transmission rate (**c**), and integrity value (**d**) of hydrogel films. * *p*-value < 0.05.

In general, wound dressing materials should have properties such as being capable of absorbing excess exudate, promoting a moist wound environment, allowing for gaseous exchange, keeping their shape when exposed to wound exudates, and being antimicrobial/antifungal [6,8,9,10]. Thus, the evaluation of fluid uptake ability, water retention capacity, water vapor transmission rate, and integrity value are those important terms that were tested to find a good candidate for wound dressing materials. Fluid uptake ability is an important parameter of a hydrogel when subjected to wound healing applications, during which the absorption of the excess wound exudates and fluids is mandated. In this study, fluid uptake ability of LMP/gelatin/CMC hydrogel films was evaluated by incubating in PBS at 37 °C. Figure 2a shows the kinetics of fluid uptake of hydrogel films. The same kinetics of a fluid uptake pattern of all hydrogel films was observed. The equilibrium state of fluid uptake was achieved after 2 h. Interestingly, fluid uptake of all hydrogel films except F-Glu-Ca increased to approximately 90%. In the period of 2 to 8 h, F-Glu-Ca film showed significantly lower fluid uptake than other formulations. From the point of hydrogel film strength, poor mechanical properties associated with a rigid polymer network might lead to a low fluid uptake ability. Importantly, high fluid uptake (%) was obtained when glycerin, acting as a humectant, was added to film formulations (F-Glu-Ca-G10, 20, 30, and 40). However, there was no significant difference between hydrogel films containing 30–40% glycerin in their ability to absorb fluid, providing those films reached their early saturation in fluid uptake at 2 h, as illustrated in Figure 2a. 

The extent of water loss from the hydrogel films when exposed to the air was evaluated with respect to their water retention capacity, as shown in Figure 2b. It is clear from this study that these materials will lose their water content when exposed to air under dry conditions over long periods of time. Our findings showed that hydrogel films, prepared with more amounts of crosslinker, demonstrated different percentages of water retention capacity. The hydrogel film with two crosslinkers (F-Glu-Ca film) exhibited greater water retention capacity when compared to the hydrogel film containing only one crosslinker (F-Glu film). This can be explained on the basis of the fact that macromolecular chains of the hydrogel film with Glu and CaCl_2_ as crosslinkers are comparatively rigid or less flexible. As compared with F-Glu film, glycerin in F-Glu-Ca-G10-40 films helped retain more water within the polymer network. Water retention capacity evaluated over a period of 24 h showed no significant difference among F-Glu-Ca films containing 10–40% glycerin. Hydrogel film with a rigid structure takes up less amounts of fluid, but demonstrates greater percentage of water retention capacity [37].

In addition to water retention, water loss from open wounds is related to the water permeability of the dressing materials. An ideal wound dressing must be able to control water loss from a wound at an optimal rate to prevent excessive dehydration [38]. The ideal range for WVTR of wound dressings has been extensively discussed and still remains controversial. Queen et al. recommend a rate of 2000 to 2500 g/m^2^/day as the WVTR without dehydration of the wound [38], whereas Xu et al. also suggest a WVTR range of approximately 1800–2300 g/m^2^/day [39]. Figure 2c shows the WVTR of the hydrogel films when placed in a moisture rich environment. The hydrogel films in the present study showed WVTR values of 1963, 1879, 1832, 1889, and 1886 g/m^2^/day for F-Glu-Ca, F-Glu-Ca-G10, G20, G30, and G40, respectively. These WVTR values were close to the range recommended for maintaining a proper fluid balance in order to sufficiently keep the wound moist. Our findings were possibly explained by the association of WVTR with a polymer network comprised of the strong bonding interactions and the presence of a humectant in those hydrogel films.

One problem of the water-swellable hydrogels is the lack of structural integrity after being hydrated and their fragile nature when exposed to wound exudates and body fluids. Thus, the integrity value of our hydrogel films was measured after 24 h soaking in PBS. The result found that only F-Glu film had lost its shape and exhibited an integrity value of 75.12%. However, other hydrogel films maintained their dimensions in PBS. The integrity of F-Glu-Ca, F-Glu-Ca-G10, G20, G30, and G40 was 89.66%, 84.68%, 84.51%, 86.42%, and 84.28%, respectively, showing no significant difference among them, as shown in Figure 2d. In 2016, Lee et al. prepared a silk fibroin nanoparticle hydrocolloid dressing and determined an integrity value in comparison with standard dressings such as Neoderm^®^. They found that when Neoderm^®^ was placed in water, it lost its shape and the shape integrity of Neoderm® was measured at values of 72%. However, their developed hydrocolloid dressing exhibited good structural integrity with an integrity value of more than 80% [26]. In our investigation, hydrogel films crosslinked by Glu and CaCl_2_ provided significantly higher integrity values when compared to hydrogel films crosslinked by Glu alone. Therefore, addition of multiple chemical crosslinkers is recommended when water-soluble hydrophilic polymeric materials, such as LMP, gelatin, and CMC, are used. 

### 3.4. Anti-Bacterial Activity of Hydrogel Film Loaded with Povidone Iodine

Our invented hydrogel films containing 10% povidone iodine (PI) clearly exerted an antimicrobial effect against the growth of *S*. *Aureus,* as shown in Table 3. During film preparation, the full-strength PI was added in order to make a final concentration of 10% by weight. In the disc diffusion test, we loaded 10 µL of 10% PI per Whatman disc because our preliminary optimization showed that 10 µL of PI solution made the paper disc saturated perfectly, thus making the research method more easily to control in each batch. It is very likely that the amount by weight in each prepared hydrogel film may contain less PI when compared with the positive control, thus showing a smaller inhibition zone. Our data suggested that the fabricated films conferred the ability to restrain an aseptic. Further study on povidone iodine release from the films might be helpful in explaining pharmacodynamics of such films containing 10% povidone iodine when used clinically. Moreover, PI at a concentration of 10% was chosen in order to compare it with commercial PI products which are also composed of povidone iodine USP (United States Pharmacopeia) 10% w/v. Although antimicrobial agents may reduce the colonization of microbes on a non-infected wound, there is little clear evidence of the effect of this on wound healing. It is likely that proper wound healing is promoted when the wound has minimized bacterial contamination. Topical use of PI is thus beneficial for the type of wound that is heavily contaminated by numbers of microbial species [40]. Our in vitro antibacterial activity against *S*. *aureus* of the prepared film containing 10% PI has suggested the film’s potential use as an antibiotic delivery vessel advantageous to the wound healing process in polymicrobial wound infections. However, care must be taken since iodine-based dressings are not recommended for use in neonates [41] and a prolonged usage of iodine leads to hyperthyroidism [42]. Remarkably, invention of this hydrogel film could be a prototypical, local drug delivery model that might be a useful medical tool, if incorporated with antibiotics during further development, for the remedy of cutaneous wounds or dermatological disorders associated with microbial infections depending on the spectrum of microorganisms targeted by loaded antibiotics. Nonetheless, whether the fabricated hydrogel films reported here are clinically effective remains to be investigated.

## 4. Conclusions

One of the most challenging issues in the dressing of cutaneous wounds is searching for the ideal material to use that could display some key attributes, i.e., ability of the films to remain intact and resist tensile stress caused by fluid intake during which the drug is locally delivered. Here, we reported an improvement of physical properties of a film formulation consisting of hydrophilic materials using multiple crosslinkers. In this study, LMP/gelatin/CMC hydrogel films were developed. Glycerin was added into film formulations to improve the mechanical properties. F-Glu-Ca-G30 film formulation, which contained 30% w/w glycerin, exhibited optimal mechanical properties, fluid uptake ability, water retention capacity, water vapor transmission rate, and integrity value. Incorporation of povidone iodine into F-Glu-Ca-G30 formulated film conferred its antibacterial property. Based on the present study, we concluded that LMP/gelatin/CMC hydrogel film loaded with povidone iodine has a potential use in biomedical applications. Considering the ability of LMP/gelatin/CMC hydrogel film to dispatch an antibiotic agent, the invention of this film might be an alternative strategy employed as a vehicle for antibiotic/aseptic delivery in the treatment of infected moist wounds. 

## Figures and Tables

**Table 1:** Compositions of low methoxyl pectin (LMP)/gelatin/carboxymethyl cellulose (CMC) films.

Sample Code	LMP (%)	Gelatin (%)	CMC (%)	Glycerin (%)	Povidone Iodine	Crosslinking Agent	
						Glu	CaCl_2_
F-Glu	1	1	1	-	-	+	-
F-Glu-Ca	1	1	1	-	-	+	+
F-Glu-Ca-G10	1	1	1	10	-	+	+
F-Glu-Ca-G20	1	1	1	20	-	+	+
F-Glu-Ca-G30	1	1	1	30	-	+	+
F-Glu-Ca-G40	1	1	1	40	-	+	+
F-Glu-Ca-G30-PI	1	1	1	30	10	+	+

**Table 2:** Thickness, tensile strength, elongation, and Young’s modulus values of hydrogel films.

Hydrogel Film	Thickness (mm)	Tensile Strength (MPa)	Elongation at Break (%)	Young’s Modulus (N/cm^2^)
F-Glu	0.30 ± 0.07 ^a^	715.73 ± 33.99 ^a^	9.43 ± 0.26 ^a^	8814.52 ± 163.07 ^a^
F-Glu-Ca	0.35 ± 0.05 ^a^	853.06 ± 52.49 ^b^	3.22 ± 0.24 ^b^	56,781.02 ± 813.06 ^b^
F-Glu-Ca-G10	0.33 ± 0.04 ^a^	730.01 ± 98.11 ^a^	23.82 ± 2.91 ^c^	6494.36 ± 366.63 ^c^
F-Glu-Ca-G20	0.34 ± 0.03 ^a^	718.39 ± 41.97 ^a^	27.07 ± 1.96 ^c^	5707.38 ± 220.05 ^c^
F-Glu-Ca-G30	0.37 ± 0.06 ^a^	547.64 ± 77.53 ^c^	32.80 ± 1.14 ^d^	2926.71 ± 146.18 ^d^
F-Glu-Ca-G40	0.37 ± 0.09 ^a^	597.16 ± 31.13 ^c^	36.25 ± 4.47 ^d^	2835.37 ± 203.49 ^d^

For each test, means with the same letter are not significantly different. Thus, means with the different letter, e.g., “a” or “b” are statistically different (*p*-value < 0.05).

**Table 3:** Antibacterial activity of hydrogel films against *S. aureus* growth.

Test Sample	Diameter of Inhibition Zone (mm)
10% Iodine solution F-Glu-Ca-G30-PI F-Glu-Ca-G30	99.23 ± 0.71 ^a^ 22.06 ± 3.44 ^b^ ND ^c^
DMSO	ND ^c^

Reported diameter of inhibition zone was derived from one of three experiments yielding similar readings. Means with the different letter, e.g., “a”, “b”, or “c” are statistically different at *p*-value < 0.05. ND indicates that the inhibition zone was not detected. Statistical analysis was performed using one-way ANOVA with Tukey’s post-hoc test for multiple comparisons.
